# Surgical Management of Compound Odontoma Associated with Unerupted Tooth 

**DOI:** 10.1155/2015/902618

**Published:** 2015-06-25

**Authors:** Andrea Pacifici, Daniele Carbone, Roberta Marini, Luciano Pacifici

**Affiliations:** Department of Oral and Maxillofacial Sciences, “Sapienza” University of Rome, Via Caserta 6, 00161 Rome, Italy

## Abstract

Odontomas represent the most common type of odontogenic benign jaws tumors among patients younger than 20 years of age. These tumors are composed of enamel, dentine, cementum, and pulp tissue. According to the World Health Organization classification, two distinct types of odontomas are acknowledged: complex and compound odontoma. In complex odontomas, all dental tissues are formed, but appeared without an organized structure. In compound odontomas, all dental tissues are arranged in numerous tooth-like structures known as denticles. Compound odontomas are often associated with impacted adjacent permanent teeth and their surgical removal represents the best therapeutic option. A case of a 20-year-old male patient with a compound odontoma-associated of impacted maxillary canine is presented. A minimally invasive surgical technique is adopted to remove the least amount of bone tissue as far as possible.

## 1. Introduction

According to the 2005 World Health Organization classification, odontoma is an odontogenic benign tumor of the young age [1]. Despite this, odontomas are clinically considered as tumor-like formations (hamartomas of dental tissues) or developmental anomalies, rather than true odontogenic neoplasms [2]. Two main types of odontoma are described: (a) complex odontoma, an amorphous and disorderly pattern of calcified dental tissues, and (b) compound odontoma, multiple miniature or rudimentary teeth [3–7]. The compound odontoma has predilection toward the anterior maxilla (61%), whereas only 34% of complex odontomas occur in this area; the complex type shows a predilection for the posterior jaws (59%) and lastly the premolar area (7%). Both variants are made of all dental tissues such as enamel, dentin, cementum, and pulp [7, 8].

Compound odontomas have numerous tooth-like structures (with altered size and shape) known as denticles. At X-ray evaluation, compound odontomas appear as well delimited lesions with a radiotransparent halo containing radiodense zones which represent small denticles, separated by fibrous septae, while in the complex types the radiodense elements appear as irregular and disorderly masses with no similarity to dental structures [2, 9]. These lesions are often associated with impacted permanent teeth [10, 11]. Impaction has been defined as the prevention of the eruption of a tooth to the expected times into a normal functional position due to the presence of an obstacle or reasons of different nature [8, 12]. In all cases, surgical removal represents the best therapeutic option and the prognosis after treatment is very favorable, with very low recurrence's incidence [7, 8, 12–17].

The aim of this case report is to describe a minimally invasive surgical procedure to remove a compound odontoma localized in the premaxilla area associated with an unerupted permanent maxillary canine. The purpose of this technique is to preserve as much as possible the surrounding bone tissue in order to promote healing and cause less discomfort to the patient during postoperative time.

## 2. Case Description

**Figure 1:**
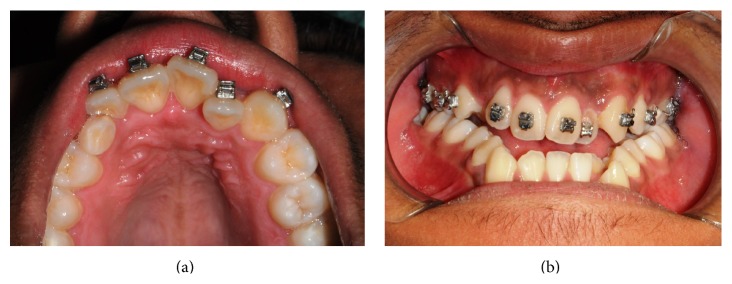
Intraoral examination (frontal and occlusal view).

A 20-year-old male patient in apparently good health conditions was referred to the Odontostomatological Clinic Unit, Department of Oral and Maxillofacial Sciences (“Sapienza” University of Rome, Italy), by his orthodontist for the absence of the right upper permanent canine. The subject had no significant medical history and had not reported oral trauma or infections. Intraoral examination showed the presence of the primary right canine over the physiological period of exfoliation, which meant no correspondence between chronological and dental ages (Figures 1(a) and 1(b)).

**Figure 2:**
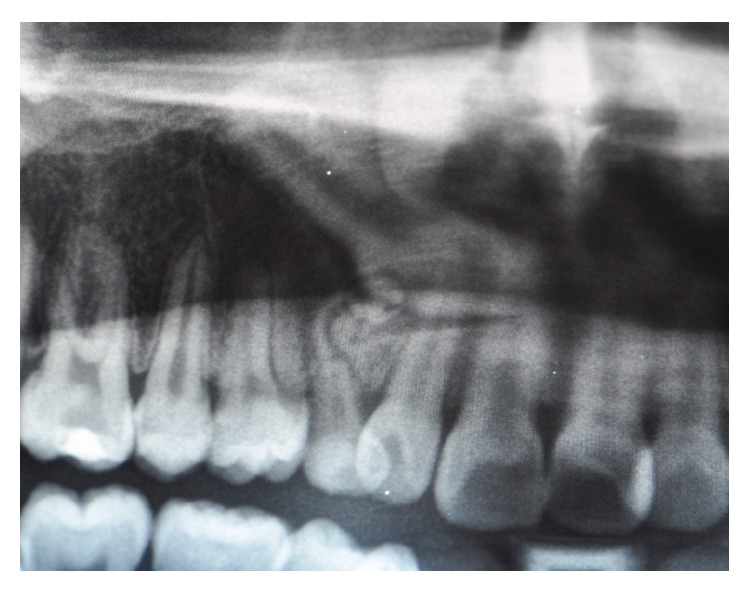
Panoramic X-ray (detail).

A radiographic examination (panoramic X-ray) showed multiple radiopaque structures compatible with a provisional diagnosis of compound odontoma and the unerupted right canine in mesial standing (Figure 2).

**Figure 3:**
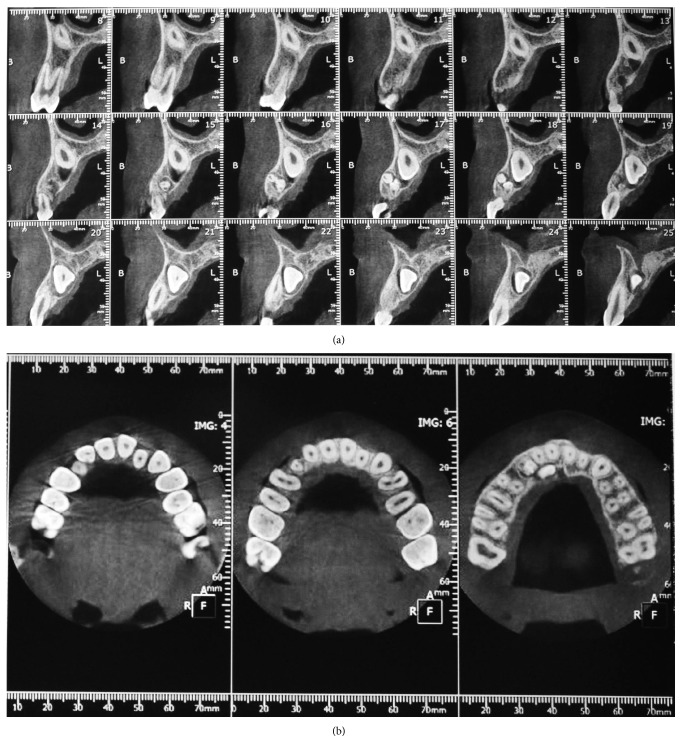
CT Dentascan: sagittal and axial view.

A computed tomography (CT) with the Dentascan program (Siemens Rs Somaton Volume Zoom Kv 120 mA 140; Siemens, Erlangen, Germany) was performed in order to define the extension of the lesion and the anatomical topography, showing the unerupted permanent tooth orally positioned compared to odontoma (Figures 3(a) and 3(b)).

**Figure 4:**
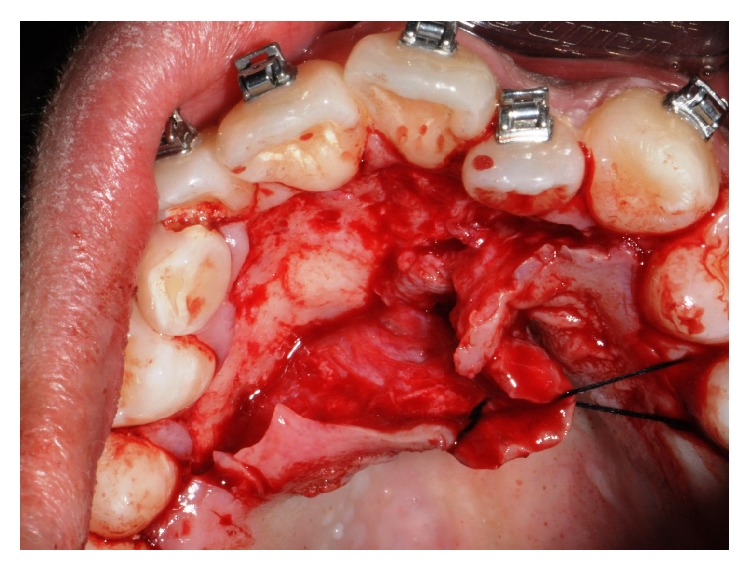
Mucoperiosteal flap reflection and bone removal.

**Figure 5:**
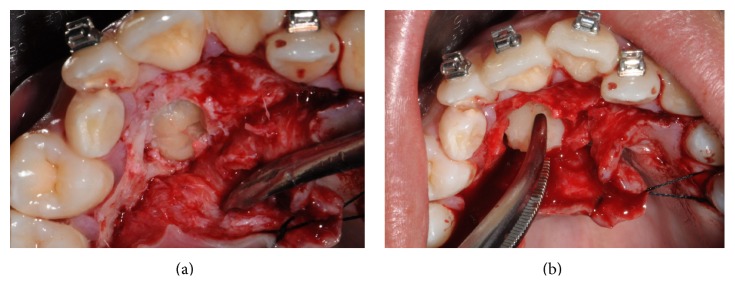
Permanent canine extraction.

**Figure 6:**
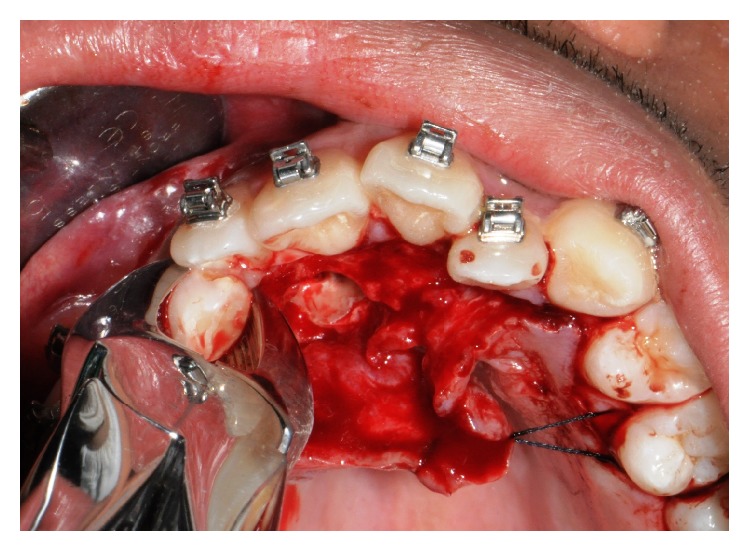
Deciduous canine extraction.

**Figure 7:**
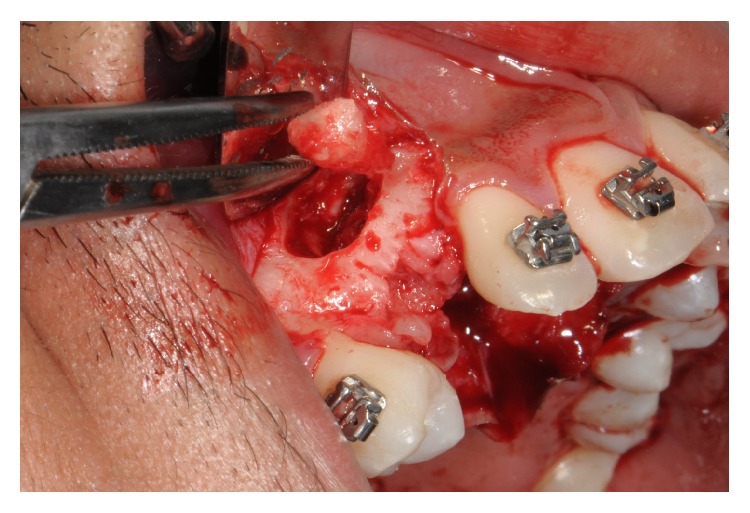
Surgical removal of compound odontoma.

**Figure 8:**
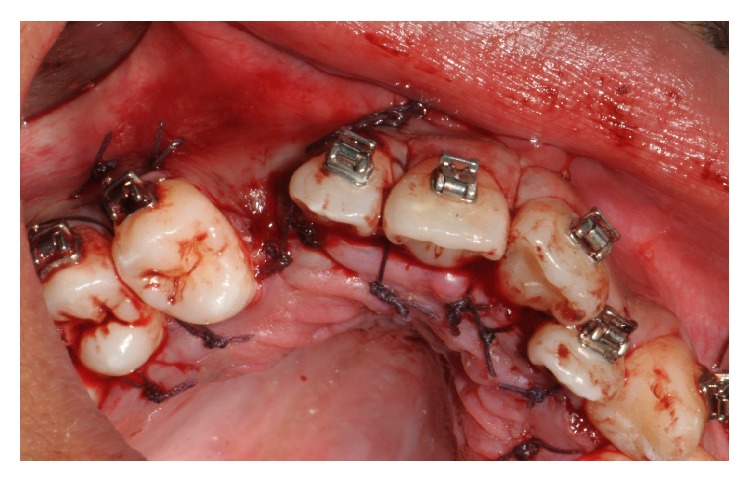
The flap repositioned and sutured.

In accordance with the patient and his orthodontist, surgical removal of the odontoma and the associated impacted canine was planned. The patient received a single dose of 2 g of amoxicillin and clavulanic acid 1 h before surgery. Surgery was performed under local anesthesia (2% mepivacaine with 1 : 100,000 epinephrine). A mucoperiosteal flap was etched and raised and bone was removed on vestibular side using a low-speed dental hand-drill and a tungsten carbide bur until the crown of the permanent impacted canine was exposed (Figure 4). After separation of the crown from the root using a high-speed dental hand-drill and a diamond bur, the tooth and its follicular sac were extracted (Figures 5(a) and 5(b)). The extraction of deciduous canine was also performed (Figure 6). A second flap was performed on the vestibular side for the extraction of single structures of the odontoma (Figure 7). The wound was carefully irrigated with physiological solution and cleaned with a sterile dressing; the flap was repositioned and sutured with 3.0 absorbable suture (Vicryl, Johnson & Johnson, Sint-Stevens-Woluwe, Belgium) (Figure 8).

The histological examination confirmed the clinical and radiographic diagnosis of compound odontoma.

The postoperative period was uneventful. Postoperative treatment consisted of amoxicillin and clavulanic acid (1 g twice a day for 5 days), paracetamol (500 mg twice a day for 2 days, and then as needed), and digluconate chlorhexidine (CHX, 0.2%) spray.

The patient was referred to the orthodontist to continue the treatment and subsequently the surgery will be programmed for implant placement.

**Figure 9:**
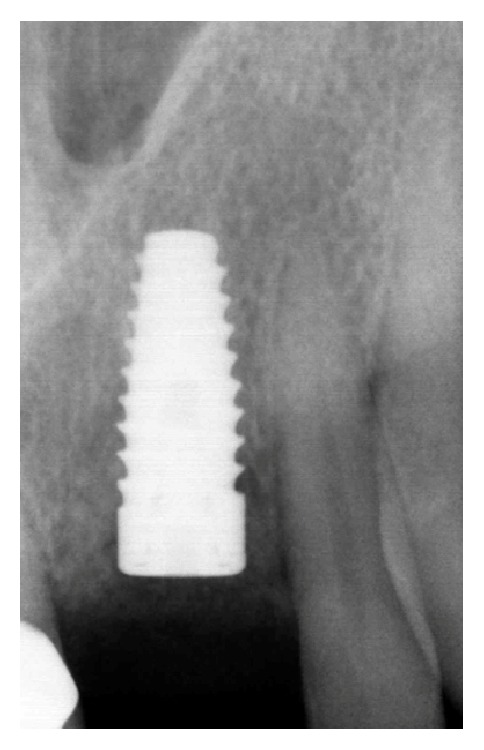
Radiographical control at 2-year follow-up.

After 2 years, the implant rehabilitation was planned: the implant (BioHorizons implant, BioHorizons Inc., Birmingham, Alabama) was placed and traditional two-stage loading protocol was adopted. Periapical radiographs taken at the time of implant placement showed no signs of recurrence or complications at the surgical site (Figure 9). After three months, the definitive prosthetic restoration will be performed with a cemented porcelain-fused-to-metal single-unit crown.

We confirm that we have read the Helsinki Declaration and have followed the guidelines concerning this report.

## 3. Discussion

The term “odontoma” was introduced by Paul Broca in 1867 to describe “tumors formed by the overgrowth of transitory or complete dental tissues.” Odontomas are intraosseous lesions mainly located in the anterior maxilla and anterior mandible, although lesions localized in gingival soft tissues have also been reported [7, 16]. The majority of odontomas are asymptomatic, although swelling, pain, suppuration, bony expansion, and displacement of teeth have been rarely observed. Their pathogenesis has been associated with a number of causes including trauma during primary dentition [9, 17], hereditary anomalies such as Gardner's syndrome, Hermann's syndrome, and basal cell nervous syndrome, odontoblastic hyperactivity, or alterations of the genetic components responsible for controlling dental development [8, 16]. The development of the odontoma is commonly associated with eruption failure of permanent teeth, impaction, and delayed exfoliation of primary teeth [17–21]. In this case, the presence of odontoma prevented the physiological eruption of permanent maxillary canine. In accordance with the literature, the patient had no pain but the lesion's pathogenesis resulted unknown: there were no reported previous traumatic or infective episodes and medical history was negative.

The treatment of choice for compound odontomas is surgical removal, followed by histopathological analysis to confirm the diagnosis [7, 22–25]. According to the literature, the optimal management of the impacted tooth should allow its conservation and repositioning in the arch [22–24]. On the other hand, impacted teeth are frequently reported to be extracted simultaneously with the odontoma [25]. In this case, the permanent canine was not retrievable and therefore it was removed together with the compound odontoma in order to rehabilitate the patient with an implant-supported prosthesis.

In this case, the removal of odontoma was followed from the extraction of the deciduous and permanent canines. The surgical difficulty was determined by necessity to adopt a technique as much conservative as possible in prediction of the subsequent intervention of implant insertion. Therefore, the bone tissue removal around the wound was minimized, making the surgical steps of lesion and tooth removal more complicated.

A careful evaluation with panoramic and CT Dentascan X-rays revealed a buccal position of the permanent canine to the odontoma. For this reason, a double access was chosen, creating two small bone gaps with the use of low-speed dental hand-drill and a tungsten carbide bur to extract the components from two different small sites instead of a single large one. This conservative approach allowed saving precious bone ridge and avoiding the formation of tissue defects; therefore it was not necessary to use filler materials or to perform guided bone regeneration procedures unlike other cases reported in the literature [25].

A histological analysis was finally taken in order to confirm the odontoma's diagnosis.

In conclusion, the presence of odontoma in association with the impacted canine needs an early diagnosis and a surgical removal treatment. A careful knowledge and an excellent evaluation of X-ray documents are essentials to resolve adequately each clinical case. The adoption of a conservative surgical approach is advisable, in order to preserve the dental tissues and obtain optimal tissue healing. A histological evaluation is necessary to confirm the correct diagnosis of odontoma.
